# Supplementary material for: Scalable modular design of solid oxide fuel cell systems for enhanced large-scale power generation
Source: Nat Commun. 2026 Feb 6;17:2421. doi: 10.1038/s41467-026-69110-y (PMC12988180; doi:10.1038/s41467-026-69110-y)
Supplement: Supplementary file 1 — Supplementary Information [file 41467_2026_69110_MOESM1_ESM.pdf]

Supplementary Information

Scalable Modular Design of Solid Oxide Fuel Cell Systems for Enhanced Large-Scale Power Generation

Xinyi Wei[a,b,*], Arthur Waeber[a], Shivom Sharma[a], Hangyu Yu[b], Jan Van herle[b], Francois Marechal[a]

[a]Industrial Process and Energy Systems Engineering-École Polytechnique Fédérale de Lausanne (EPFL), Sion, CH-1950, Valais, Switzerland
[b]Group of Energy Materials-École Polytechnique Fédérale de Lausanne (EPFL), Sion, CH-1950, Valais, Switzerland

*Corresponding author E-mail: xinyi.wei@epfl.ch; xinyiwei.epfl@gmail.com

A. Supplementary Method

A1. Pressure drops calculations

Pressure drop is a critical factor in evaluating the efficiency of the proposed hybrid design. While a conventional equation is used for the stack (as cited in the main article), the methods and assumptions for estimating pressure drops in the reformer and burner are detailed below.

A1.1 Pre-reformer pressure drop

The pressure drop for the pre-reformer is estimated using the Ergun equation, which describes fluid flow through packed beds. A Monte Carlo simulation with 10,000 iterations accounts for variability in gas hourly space velocity (GHSV) and the reactor length-to-diameter (L/D) ratio. A nominal flow rate of 0.145 m^3/h per cell is used, with fluid properties such as dynamic viscosity and density obtained from Aspen Plus. The reformer is modelled as 48 identical parallel tubes, as referenced in the main article. In each iteration, reactor dimensions are recalculated based on sampled GHSV and L/D values while maintaining a constant total reactor volume to meet the specified flow conditions.

The bed void fraction (ε) was estimated using Equation 1, based on the total catalyst surface area A_{cat}, particle diameter d_p, and tube bed volume V_{bed}. Since the pre-reformer is designed to convert only 15–20% of the incoming methane, the resulting bed void fraction is relatively high, approximately 0.85.

$$\varepsilon = 1 - \frac{A_{\text{cat}} d_p}{6 V_{\text{bed}}} \tag{1}$$

The superficial gas velocity was calculated for each sampled configuration. The pressure drop across the bed was then computed using the Ergun equation.

$$\Delta P = \frac{L}{d_p}\left(\frac{150(1-\varepsilon)^2 \mu v}{\varepsilon^3 d_p} + \frac{1.75(1-\varepsilon)\rho v^2}{\varepsilon^3}\right) \tag{2}$$

Where:

- ΔP is the total pressure drop (Pa),
- L is the bed length (m),
- d_p is the particle diameter (m),
- μ is the dynamic viscosity (Pa·s),
- ρ is the gas density (kg/m^3),
- v is the superficial gas velocity (m/s),
- ε is the void fraction (–).

This method provides a rough estimate of the pressure drop in the pre-reformer, which is crucial for computing the electrical efficiency of the proposed hybrid design. Some assumptions and limitations are listed below.

- The catalyst bed consists of uniformly sized spherical particles (3 mm diameter).
- The flow is steady, isothermal, and incompressible.
- Pressure drop contributions from inlet/outlet manifolds and tube bends were neglected.
- All tubes were assumed to operate under identical flow conditions.

The mean and standard deviation of the pressure drop distribution are calculated based on the full Monte Carlo sample set. The estimated pressure drop may be chosen as the maximum value achieved.

A1.2 Burner pressure drop

The total pressure drop for the burner was estimated by accounting for both distributed (frictional) and localized (singular) losses. A Monte Carlo simulation with 10,000 iterations was performed to capture uncertainties in gas hourly space velocity (GHSV), burner length-to-diameter (L/D) ratio, and the total singular loss coefficient (K_{sing}), which represents pressure drops due to abrupt geometry changes. The total volumetric flow rate, dynamic viscosity, and inlet gas density were obtained from Aspen Plus. For each iteration, burner dimensions were recalculated based on sampled GHSV and L/D values while conserving volumetric flow. The Reynolds number for each case was computed (Equation 3).

$$Re = \frac{\rho v D}{\mu} \tag{3}$$

Where:

- ρ is the gas density (kg/m³),
- v is the superficial velocity (m/s),
- D is the burner diameter (m),
- μ is the dynamic viscosity (Pa·s).

The friction factor f was estimated depending on the flow regime, as given below.

- For laminar flow (Re < 2000), f = 64/Re
- For transitional and turbulent flow (Re > 2000), the Blasius correlation was applied, $f = \frac{0.079}{Re^{0.25}}$

The distributed pressure drops, namely friction and localized, are computed using the Darcy–Weisbach equation (Equations 4 and 5).

$$\Delta P_{\text{frictional}} = f \frac{L}{D} \frac{\rho v^2}{2} \tag{4}$$

$$\Delta P_{\text{localized}} = K_{sing} \frac{\rho v^2}{2} \tag{5}$$

Due to a strong uncertainty in the estimated pressure drop for burner, the estimated pressure drop is chosen with a safety factor of 3. Some assumptions and limitations are listed below.

- Steady-state, isothermal, and incompressible gas flow.
- Wall roughness or entrance effects were not explicitly modelled beyond the assigned K_{sing}.
- The burner cross-section is assumed to be perfectly circular.
- There are no-slip conditions at walls, and uniform velocity profiles at the inlet were assumed.

A2. Log-log regression for component cost functions

The cost functions were estimated using open-access data coming from Battelle, a global research and development organization, and supported by the U.S Department of Energy. The report (see reference [32][38] in the main body) provided data on 6 different scales and 4 different annual production volume and the log-log regression were computed using python.

(a) (b)

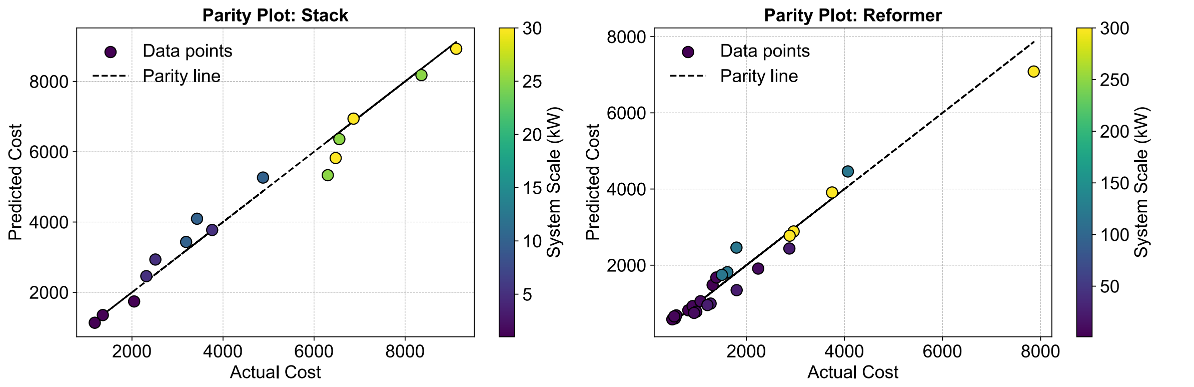

Supplementary Figure A2.1: Log-log regression parity plot for: (a) stack and (b) reformer modules. Source data are provided in 'Source Data.xlsx'.

(a) (b)

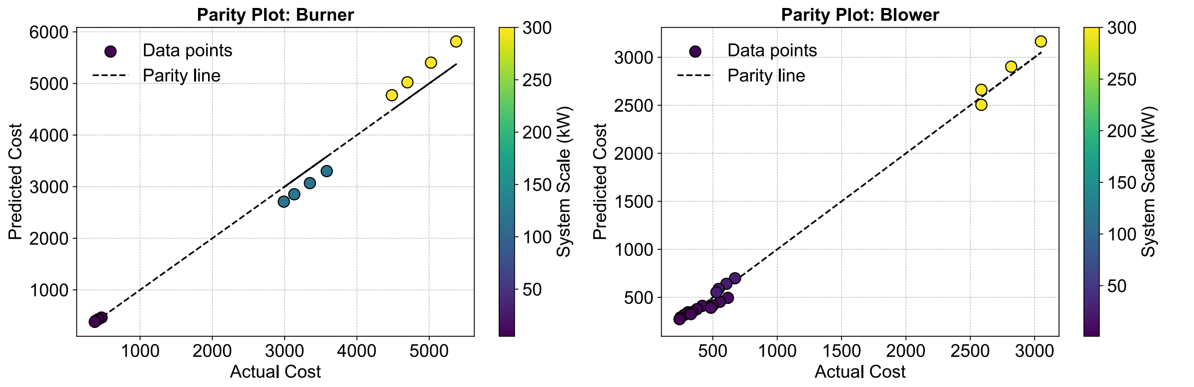

Supplementary Figure A2.2: Log-log regression parity plots for: (a) burner and (b) blower modules. Source data are provided in 'Source Data.xlsx'.

(a) (b)

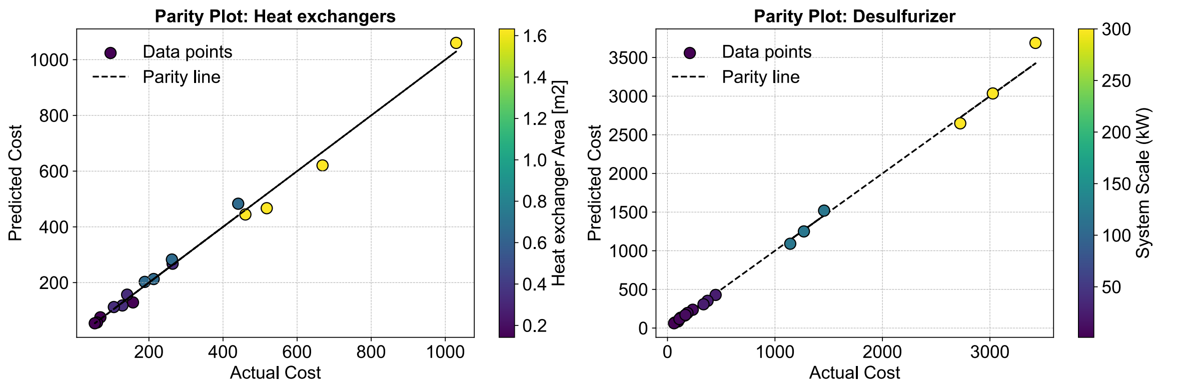

Supplementary Figure A2.3: Log-log regression parity plots for: (a) heat exchangers and (b) desulfurizer modules. Source data are provided in 'Source Data.xlsx'.

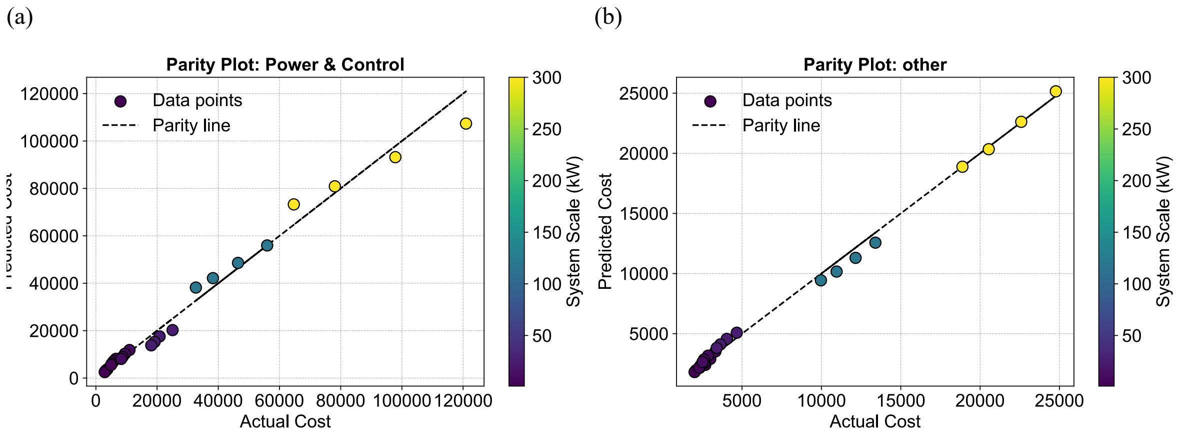

Supplementary Figure A2.4: Log-log regression parity plots for: (a) power and control module and (b) other BoP components. Source data are provided in 'Source Data.xlsx'.

Equation 6 estimates cost based on the component size and the number of units produced per year. Supplementary Table A2.5 presents the various components empirical coefficient values.

$$\log(C_{cp}) = a + b \cdot \log(P) + c \cdot [\log(P)]^2 + d \cdot \log(N) + e \cdot [\log(N)]^2 \quad (6)$$

Supplementary Table A2.5: Empirical parameters of the cost functions for each component module

Equipment	P (Range, Unit)		N (Range, Unit)		a	b	c	d	e
	Validity Range	Unit	Validity Range	Unit					
Stack	[1 - 30]	kW (SOFC)	[1k- 50k]	Units/yr	8.2174	0.4807		-0.1093	
Reformer	[0.4 - 120]	kg/h	[100 - 50k]	Units/yr	9.3620		0.0481	-0.5739	0.0274
Burner	[9.1 - 550]	kg/h	[100 - 50k]	Units/yr	5.2922	0.6175		-0.0318	
Blower	[0.065 - 0.8]	m^3/s	[1k - 50k]	Units/yr	6.0168		0.0681	-0.0376	
Compressor	[0.065 - 0.8]	m^3/s	[1k - 50k]	Units/yr	6.4868		0.0681	-0.0380	
Desulfurizer	[0.4 - 120]	kg/h	[1k - 50k]	Units/yr	5.0462	0.2881	0.0648	-0.0847	
Heat exchangers	[0.14 - 1.6]	m^2	[100 - 50k]	Units/yr	8.3680	0.8636		-0.5055	0.0237
Power and Controls	[1 -300]	kW (SOFC)	[100 - 50k]	Units/yr	8.5023	0.4454	0.0253	-0.0615	
Others	[1 -300]	kW (SOFC)	[100 - 50k]	Units/yr	7.9975		0.0722	-0.0461	

Supplementary Table A2.6: Summary of distribution function for each parameter used in uncertainty analysis. $n(\mu, \sigma)$ and $u(V_{min}, V_{max})$ are the normal and uniform distributions, respectively. σ is expressed as a percentage of μ. 1,000 simulation runs were used.

Parameter	Unit	Distribution function
CAPEX low TRL Components	$	$n(CAPEX_m, 20\%)$
CAPEX high TRL Components	$	$n(CAPEX_m, 10\%)$
Lifetime low TRL Components	Years	$u(5, 12)$
Lifetime high TRL Components	Years	$u(5, 12)$
Discount rate	%	$n(5, 10\%)$
Fuel price	Cents/kWh	$n(10, 10\%)$

B. Supplementary Results

B1. Heat flow for different system designs/configurations

Supplementary Table B1.1: Heat flow for hybrid/FCFA, NCNA and NCHA designs

**Hybrid design or FCFA (50 kW)**			
H1	25	103	0.278
H2	25	103	0.512
H3	103	103.1	3.440
H4	103.1	539	2.270
H5-1	539	539.1	0.461
H5-2	539	539.1	0.461
H6-1	539.1	680	0.441
H6-2	539.1	680	0.441
H15	533	533.1	0.429
H25	518	518.1	0.428
H35	519	519.1	0.391
CSH1	35	680	42.756
CSH2	25	606	36.242
CSC1	901	95	15.484
CSC2	95	25	13.013
**NCNA (50 kW)**			
H1	25	103	0.297
H2	25	103	1.281
H3	103	103.1	8.600
H4	103.1	539	5.674
H5	539	539.1	2.305
H6	539.1	680	2.206
H7	35	680	106.908
C1	901	95	28.085
C2	95	25	19.694
C3	750	25	116.878
**NCHA (50 kW)**			
H1	25	103	0.289
H2	25	103	0.255
H3	103	103.1	1.714
H4	-	-	-
H5	548	548.1	1.906
H6	548.1	680	2.993
H7	35	680	63.809
C1	901	95	11.163
C2	95	25	6.784
C3	750	25	70.158

B2. Pressure drops

Supplementary Table B2.1: Summary of all input and output parameters of Monte-Carlo simulations for the pre-reformer and the burner. The GHSV and L/D ratio are both varied according to a uniform distribution in the form $U(min, max)$.

Quantity	Symbol	Unit	Reformer	Burner
Flow Rate	Q	m^3/h	145	108
Viscosity	μ	Pa s	3.12E-05	3.09E-05
Gas Hourly Space Velocity	GHSV	h^{-1}	$U(14k, 26k)$	$U(15k, 50k)$
Aspect Ratio	L/D	-	$U(1, 4)$	$U(9, 21)$
Pressure Drops Coefficient	K_{sing}	-	–	$U(4, 16)$
Number of Simulation Runs	N_{runs}	-	100000	100000
Mean Pressure Drop	ΔP_{mean}	mbar	4.41	6.21
Standard Deviation	ΔP_{std}	mbar	2.85	4.01
Estimated Pressure Drop	ΔP_{max}	mbar	10	15

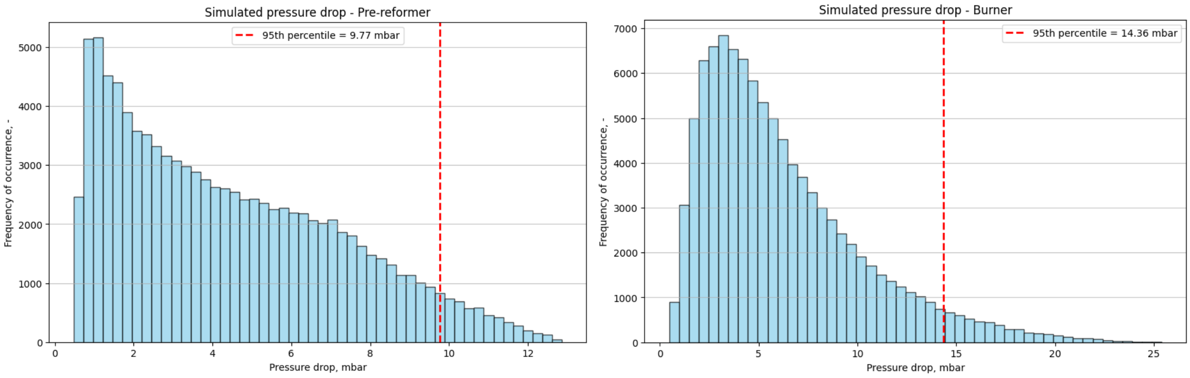

Supplementary Figure B2.2: Pressure drops for the reformer (left) and the burner (right) calculated from input parameters gathered in Table B2.1.

B3. Economic analysis

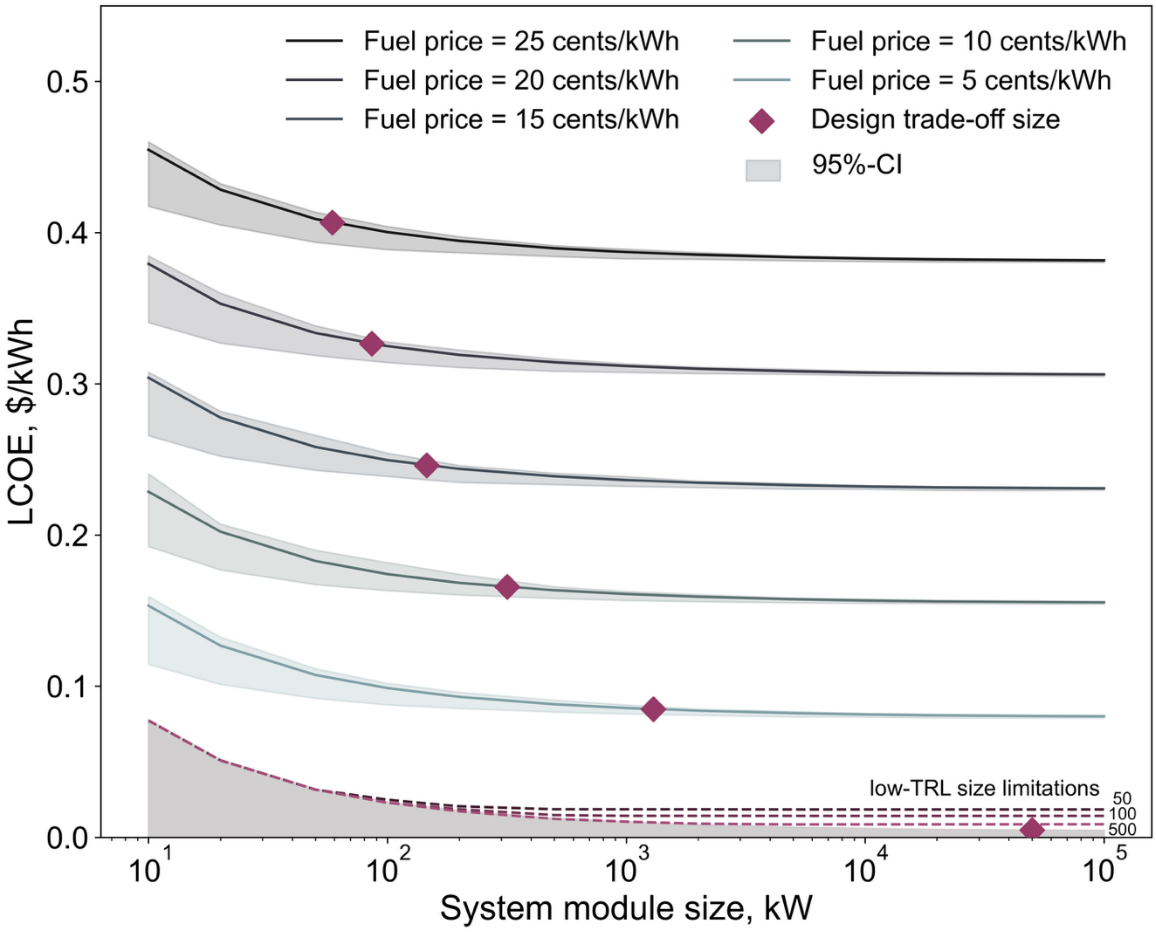

Supplementary Figure B3.1: Sensitivity analysis on the fuel price with trade-off sizes for the hybrid design strategy, including market limitations of 50, 100, and 500 kW for low TRL components. Source data are provided in 'Source Data.xlsx'.
